# Utility of ACMG classification to support interpretation of molecular genetic test results in patients with factor VII deficiency

**DOI:** 10.3389/fmed.2023.1220813

**Published:** 2023-07-14

**Authors:** Rosa Sonja Alesci, Carola Hecking, Benjamin Racké, Detlev Janssen, Carl-Erik Dempfle

**Affiliations:** ^1^IMD Blood Coagulation Centre, Bad Homburg, Germany; ^2^Institute of Immunology and Genetics, Kaiserslautern, Germany; ^3^Med-i-Scene Concept, Weisendorf, Germany; ^4^IMD Blood Coagulation Centre, Mannheim, Germany

**Keywords:** FVII deficiency, genotype, phenotype, ACMG classification, genetic variations

## Abstract

**Background:**

The American College of Medical Genetics and Genomics (ACMG) and the Association for Molecular Pathology (AMP) have introduced an internationally shared framework for variant classification in genetic disorders. FVII deficiency is a rare inherited autosomal recessive bleeding disorder with sparse data concerning ACMG classification.

**Methods:**

To develop an approach which may improve the utility of molecular genetic test results, 129 patients with FVII deficiency were retrospectively assigned to six subgroups for exploratory analysis: *F7* gene wildtype *(group 1)*, ACMG 1 (benign variant) or ACMG 2 (likely benign variant), only *(group 2)*, ACMG 3 (variant of uncertain significance) ± ACMG 1–2 heterozygous or not classified variant *(group 3)*, ACMG 4 (likely pathogenic variant), or ACMG 5 (pathogenic variant) single heterozygous ± ACMG 1–3 single heterozygous *(group 4)*, ACMG 4–5 homozygous or ≥2 ACMG 4–5 heterozygous or ≥1 ACMG 4–5 heterozygous plus either ACMG 1 c.1238G>A modifying variant homozygous or ≥2 ACMG 1–3 *(group 5)*, FVII deficiency and another bleeding disorder *(group 6)*.

**Results:**

Eleven of 31 patients (35.5%) in group 5 had abnormal ISTH-BS (*n* = 7) and/or history of substitution with recombinant factor VIIa (*n* = 5) versus 4 of 80 patients (5.0%, *n* = 1 abnormal ISTH-BS, *n* = 3 substitution) in groups 1 (*n* = 2/22), 2 (*n* = 1/29), 3 (*n* = 0/9), and 4 (*n* = 1/20). Four of 18 patients (22.2%) with FVII deficiency and another bleeding disorder (group 6) had an abnormal ISTH-BS (*n* = 2) and/or history of substitution with recombinant factor VIIa (*n* = 3).

**Conclusion:**

Patients with a homozygous ACMG 4–5 variant or with specific combinations of heterozygous ACMG 4–5 ± ACMG 1–3 variants exhibited a high-risk bleeding phenotype in contrast to the remaining patients without another bleeding disorder. This result may serve as a basis to develop a genotype/phenotype prediction model in future studies.

## Introduction

Congenital factor VII (FVII) deficiency is an autosomal recessively inherited rare bleeding disorder. The clinical phenotypes in FVII deficient patients show a wide variation from asymptomatic to severe with a poor correlation to FVII activity (1). Some patients with very low FVII activity had no bleeding symptoms whereas patients with partial FVII deficiency may have recurrent bleeding episodes (2). FVII levels above 20% are thought to be sufficient for prevention against spontaneous bleeding (1), possibly due to an enhanced effect of TF-FVII complex which activates coagulation even with small amounts of FVII (1, 3). However, previously asymptomatic patients with FVII levels above 20% may have an increased bleeding risk in situations such as major surgery or trauma. FVII deficiency may be caused by a quantitative FVII defect (type I, decreased FVII antigen) or qualitative FVII defect (type II, normal FVII antigen) (1).

Even though the prevalence of FVII deficiency was repeatedly reported to be 1:300,000–1:500,000 individuals (4–7) it is believed that mild, moderate or severe forms of FVII deficiency are by far more frequent reaching 1:59,000 individuals (8).

The coagulation factor VII gene (*F7*, HGNC ID: 3544) is located on chromosome 13 (13q34) with nine exons and eight introns, which compose a 12.8 kb gene locus near the telomeric region of the chromosome besides the gene promotor region (9). In the coagulation factor VII variant database of the European Association for Haemophilia and Allied Disorders (EAHAD) 271 unique variants of *F7* gene in 1058 individuals with FVII activity below or above the normal range were recorded until May 2022 (10). Most *F7* variants were small lesions with single nucleotide substitutions (point) in 86.7% of individuals followed by deletions (8.9%), duplications (2.6%), indel rearrangements (1.1%) and insertions (0.7%) (10). Missense variants represented 74% of single nucleotide substitutions (11).

The genotype/phenotype relationship in FVII deficient patients has extensively been studied in humans (2, 8, 12). FVII levels below 10% were seen in about 50% of patients who are homozygous or compound heterozygotes for pathogenic *F7* gene variants compared to 7% of patients with heterozygous *F7* pathogenic gene variants (6). Bleeding symptoms were discovered in 71% of patients with homozygous causative *F7* gene variants versus 50% in compound heterozygotes and 19% in heterozygotes (2).

Whereas FVII:C shows a poor correlation to severity of bleeding phenotype the type and the site of a *F7* variant may be helpful to predict hemorrhagic risks (13). For example, mutations affecting TFPI-binding exosites of FVII may markedly prolong clotting time (14). The utility of molecular genetic diagnostics depends on the validity of the pathogenicity classification for a specific variant detected. As a major step to establish an internationally shared framework for a systematic, objective and evidence-based variant classification in genetic disorders the American College of Medical Genetics and Genomics (ACMG) and the Association for Molecular Pathology (AMP) published standards and guidelines for the interpretation of sequence variants in 2015 (15). However, these guidelines lacked specificity in several areas or resulted in contradictory or ambiguous interpretations. Therefore, validated “semiquantitative, hierarchical evidence-based rules for locus interpretation” (Sherloc) was developed by the ACMG-AMP (16).

The ACMG classification uses a specific standard terminology to describe gene variants that are the cause of Mendelian disorders and is based on a complex process for assigning a variant to one of the five ACMG categories (15, 16): Variants classified as pathogenic (ACMG 5) or likely pathogenic (ACMG 4) have met specific criteria and may be used by the health-care provider for clinical decision making. A variant considered benign (ACMG 1) is not causative for the patient’s disorder or symptoms. ACMG 2 describes a variant which is likely benign (ACMG 2). Variants of uncertain significance (VUS; ACMG 3) should imply efforts for changing the classification to pathogenic or benign based on additional information.

In general, interpretation of gene variants classified as pathogenic is challenging (17). A pathogenic variant may be present in an individual without phenotypic correlate. In addition, classifications of specific variants may change over time or differ between databases such as Human Gene Mutation Database (HGMD) and ClinVar (17). To be useful as a contribution for therapeutic decisions the variants of the *F7* gene should primarily be analyzed with respect to abnormal bleeding phenotype. There are several *F7* gene variants associated with reduced FVII activity which are classified as benign (ACMG 1), for example the frequent missense *F7* variant p.(Arg353Gln), also known as Arg353Gln polymorphism (R353Q; rs6046) or described by the single nucleotide substitution of Arginine by Glycine residue in position 413, p.(Arg413Gln) or by the complementary DNA (cDNA) change c.1238G>A. This variant leads to a mean decrease of FVII:C activity by about 20–30% in individuals with M1M2 genotype (heterozygous) (18–21), and by 43% in homozygotes (20). The minor allele frequency (MAF) of 0.1265 means that about 12.7% of the population are carriers of this variant. The variant c.1238G>A is known to diminish FVII secretion and is thought to be not directly pathogenic but modifying, i.e., contributing to the consequence of pathogenic variants (11, 22), for example by augmentation the FVII deficiency caused by the p.(Ala354Val) (c.1061C>T) variant (23).

The EAHAD *F7* variant database provides tools to facilitate variant classification and has been prepared to list the pathogenicity of variants based on the ACMG classification (11). Unknown pathogenicity of a specific *F7* gene variant represents only one of the difficulties in conjunction with interpretation of molecular genetic test results. Heterozygosity may be of clinical relevance if there are two or more pathogenic variants. To differentiate between compound heterozygous patients (both alleles affected) and double heterozygous patients (two variants in the same allele) molecular genetic testing of the mother or the father is usually needed. However, this is a hurdle in clinical practice. In addition, benign *F7* gene variants may have a modifying effect in combination with pathogenic variants which increases the bleedings risk. Based on a large cohort of patients with FVII deficiency we explored an approach to increase the utility of ACMG classification which should facilitate interpretation of molecular genetic test results as a basis for successive advice and decision making in daily routine.

## Materials and methods

### Patients

Patients with FVII deficiency and genetic analyses prompted at the Coagulation Center Hochtaunus, Bad Homburg Germany, and the Coagulation Center Mannheim, Germany, between August 2012 and December 2021 were included in this retrospective exploratory analysis. According to center’s routine all patients with suspected bleeding disorder had completed the ISTH-SSC bleeding assessment tool (ISTH-BAT), a standardized questionnaire which can be used to generate a bleeding score (BS) in patients with bleedings disorders (24–26). Cut offs for an abnormal BS were ≥3 for children <12 years, ≥4 for males ≥12 years, and ≥6 for females ≥12 years. The higher score in females is due to the possibility of menorrhagia and postpartum bleeding (25). Besides standardized questionnaire the notes made by the physicians to document medical history in a non-standardized manner were used to identify and analyze bleeding symptoms. Treatment for substitution of FVII deficiency was documented.

### Coagulation tests

As both centers belong to the same company the same procedures and reagents were used for coagulation tests (FVII:C, activated partial thromboplastin time, prothrombin time (PT) with Quick value) which were performed locally by routine. FVII:C was measured using Coagulation FVII deficient plasma (Siemens Healthineers, Erlangen, Germany) and the fully automated analyzer Sysmex^®^ (Siemens Healthineers, Erlangen, Germany).

### Molecular genetic analyses

Molecular genetic analyses were performed at the Office for Human Genetics, Wiesbaden, Germany, and at the Institute for Immunology and Genetics, Kaiserslautern, Germany. Before drawing peripheral blood for genetic testing all patients had to sign an informed consent form according to the German Gene Diagnostics Act. After isolation of genomic DNA the nine exons of the *F7* gene on chromosome 13q34 and their flanking introns (since January 2015: RefSeqGene: NG_009262.1, transcript: GenBank accession number NM_000131.4) were analyzed by DNA sequencing. For exclusion of large deletions and duplications a variation of the multiplex polymerase chain reaction (PCR) named MLPA (Multiplex ligation-dependent probe amplification) analysis was performed using the MLPA-kit P207 from MRC Holland, Amsterdam, The Netherlands.

### Assessment of pathogenicity of variants

Reports of individual molecular genetic testing in the cohort were screened for information about ACMG classification of specific variants. If a variant has not been classified, for example due to molecular genetic testing before the ACMG–AMP standards and guidelines were introduced, databases as follows were screened for information on pathogenicity: GnomAD,^1^ ClinVar,^2^ VarSome,^3^ Franklin by genoox,^4^ EAHAD-CFDB Factor VII Gene (*F7*) Variant Database.^5^ Additional information was available at the EAHAD *F7* database (MAF, Grantham Score, PolyPhen-2 Prediction, SIFT Prediction, and PROVEAN Prediction) (11), and by case specific literature searches. If a variant could not be assigned to ACMG 1, 2, 4, or 5 it was classified as unknown or – if such classification was available – as variant of uncertain significance (VUS, ACMG 3).

### Statistics

The data were analyzed descriptively with categories of patients expressed as numbers and percentages. For the other variables, mean values ± standard deviation (SD) as well as median (minimum, maximum) were calculated.

A combined endpoint consisting of an abnormal bleeding score and/or substitution history for FVII deficiency was used to identify high risk phenotypes in patients without another bleeding disorder, and to support the hypothesis that heterozygous ACMG 5 variants are of clinical relevance if combined with homozygous modifying ACMG 1–3 variants.

**FIGURE 1:**
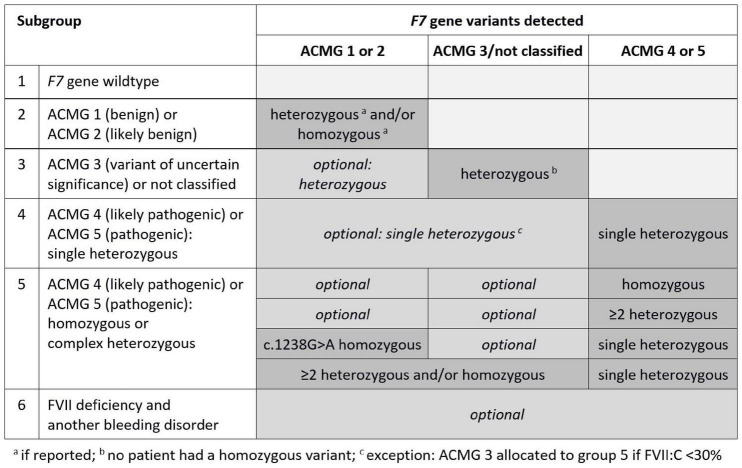
Genetic subgroups according to F7 genotype, ACMG classification and presence of another bleeding disorder.

First, a subgroup of FVII deficient patients with homozygous modifying ACMG 1 variant c.1238G>A plus heterozygous ACMG 5 variant c.1061C>T was selected and compared with a subgroup of patients who had either a single c.1238G>A variant, only, or were heterozygous for c.1238G>A variant plus heterozygous for ACMG 5 variant c.1061C>T. Thereafter, the total cohort was divided into six genetic subgroups according to *F7* genotype and presence of another bleeding disorder as shown in Figure 1. The combined endpoint mentioned above was used to compare group 5 (assumed high-risk phenotype) with pooled groups 1 to 4 (assumed low-risk phenotype).

Ethical review and approval was not required for the study on human participants in accordance with the local legislation and institutional requirements. Written informed consent from the patients was not required to participate in this study in accordance with the local legislation and institutional requirements. The retrospective analysis complied with the principles of the Declaration of Helsinki and the Good Clinical Practice (GCP).

## Results

In total, 129 patients with diagnosis of FVII deficiency (mean FVII:C 42.4% ± 15.1%) and availability of molecular genetic report were evaluated. Eighty-nine patients were females (69.0%). Mean age at molecular genetic testing was 34.1 ± 16.7 years (median 32.5 years, range 0–84 years). Reasons for consultation of the Coagulation Center were bleeding symptoms in 23 patients (17.8%), reduced Quick value in 31 patients (24.0%), testing of a family member in 12 patients (9.3%), known FVII deficiency in 17 patients (13.2%), recurrent abortions and/or wish for a child in 38 patients (29.5%), and miscellaneous in 8 patients (6.2%).

### Variants

*F7* gene wildtype (WT) was diagnosed in 25 patients (19.4%). In total, the genetic reports of 104 non-WT patients contained 182 variant findings (1.8 ± 1.2 variants per patient; median 1, maximum 10). Most findings in the molecular genetic reports were ACMG 1 or 2 variants (*n* = 102) followed by ACMG 4 or 5 variants (*n* = 65). About half of ACMG 1 or 2 findings were homozygous (*n* = 54; 53%), whereas 62 of 65 ACMG 4 or 5 findings (95.4%) were heterozygous. Further details of the variants are listed in the Supplementary Tables 1–4.

About half of the 53 different *F7* gene variants identified were pathogenic (ACMG 5 *n* = 17; 32.1%) or likely pathogenic (ACMG 4 *n* = 11; 20.8%) (Supplementary Tables 1–3). Only 13 variants were benign (ACMG 1 *n* = 10; 18.9%) or likely benign (ACMG 2 *n* = 3; 5.7%). Ten variants were of uncertain significance (ACMG 3; 18.9%), and 2 variants remained unclassified (3.8%). Twenty variants (37.7%) seen in one (*n* = 16), two (*n* = 2) or three (*n* = 2) patients are not listed in the EAHAD database for *F7* gene variants, mostly ACMG 1 or 2 variants (*n* = 8), and ACMG 3 or not classified variants (*n* = 8) (Supplementary Tables 1–3). For many variants with single nucleotide substitutions the EAHAD *F7* database contains information concerning MAF, Grantham Score, PolyPhen-2 Prediction, SIFT Prediction, and PROVEAN Prediction (Supplementary Tables 1–3).

### Frequent variants c.1238G>A ± c.1061C>T

The missense *F7* gene variant c.1238G>A was detected in 81 of 104 non-WT patients (77.9%), 47 of 81 (58.0%) were homozygous (Supplementary Table 1). In 35 of 81 carriers of c.1238G>A variant (43.2%) no other *F7* gene variant was found (Table 1). Mean FVII:C in 12 patients with single heterozygous c.1238G>A variant was 53.8 ± 14.9% with FVII:C below 50% in two patients (18 and 30%, respectively). Twenty-three patients with single homozygous c.1238G>A variant had mean FVII:C of 44.5 ± 12.1%.

**TABLE 1:** Characteristics, ISTH BS, and substitution in FVII deficient patients with c.1238G>A variant ± heterozygous c.1061C>T variant.

	Single c.1238G>A, only		c.1238G>A plus heterozygous c.1061C>T^a^	
	Heterozygous c.1238G>A	Homozygous c.1238G>A	Heterozygous c.1238G>A	Homozygous c.1238G>A
All patients	*n* = 12	*n* = 23	*n* = 6	*n* = 12
Age^c^	35.1 ± 18.6	30.7 ± 16.7	24.5 ± 8.7	32.1 ± 16.8
Female sex	8 (66.7%)	15 (65.2%)	3 (50.0%)	6 (50.0%)
FVII activity (%)^b^	53.8 ± 14.9 57.5 (18–66)	44.5 ± 12.1 45 (8–65)	38.3 ± 11.1 41 (19–53)	32.6 ± 5.8 30 (27–44)
Quick value (%)^b^	75.5 ± 13.5 79.5 (48–87)	67.6 ± 13.0 69 (21–85)	65.0 ± 11.6 60.5 (53–81)	59.3 ± 3.1 58.5 (56–66)
Patients with no other bleeding disorder, only	*n* = 8	*n* = 19	*n* = 5	*n* = 12
Female sex	6 (75.0%)	11 (57.9%)	3 (60.0%)	6 (50.0%)
**Reason for consultation**				
- Bleeding symptoms	3/8	1/19	1/5	3/12
- Laboratory findings	2/8	3/19	2/5	6/12
- Family screening	1/8	5/19	1/5	0/12
- FVII deficiency known	0	4/19	0/5	2/12
- Abortions/wish for child	1/6	4/11	1/2	1/6
- Others	1/8	2/19	0/5	0/12
**ISTH BS^c^**				
- Females	1.3 ± 1.0	1.6 ± 1.8	1.7 ± 1.5	3.7 ± 2.8
- Males	1.0 ± 1.4	1.3 ± 1.6	2.5 ± 0.7	1.3 ± 2.0
ISTH BS ≥ cut-off	0/8 (0%)	0/19 (0%)	0/5 (0%)	4/12 (33.3%)
- Children ≥ 3	0/0	0/1	0/0	2/2
- Males ≥ 4	0/2	0/7	0/2	0/5
- Females ≥ 6	0/6	0/11	0/3	2/5
rFVIIa substitution	1 (15.5%)^d^	0 (0%)	0 (0%)	2 (16.7%)^d^

BS, bleeding score; rFVIIa, recombinant factor VIIa; SD, standard deviation.

^a^Up to 8 further *F7* gene variants present (mostly c.1391Cdel, *n* = 6) in 3 patients heterozygous for c.1238G>A and in 5 patients homozygous for c.1238G>A.

^b^First row: mean ± SD; second row: median (range).

^c^Mean ± SD.

^d^All patients with rFVIIa substitution had ISTH BS below cut-off.

The grey color differentiates two groups with single c.1238G>A variants, only, from two groups with c.1238G>A variants plus c.1061C>T variant.

In 18 patients with c.1238G>A variant the ACMG 5 variant c.1061C>T was present (all heterozygous). One of these had *F7* deficiency together with another bleeding disorder (von Willebrand syndrome). The c.1061C>T variant was not accompanied by the c.1238G>A variant in one patient, only. However, this male patient with FVII:C 33% and ISTH BS 5 had the c.1388delC variant (heterozygous, ACMG 4) which was also present in six other patients together with the c.1061C>T variant. As shown in Table 1, six of 12 patients (50.0%) with homozygous c.1238G>A variant and heterozygous c.1061C>T variant had an abnormal ISTH bleeding score (*n* = 4) and/or history of rFVIIa substitution (*n* = 2) versus only 1 of 41 patients (2.4%) in the other three subgroups of patients with c.1238G>A variant. This finding supports allocation of patients with a single heterozygous ACMG 4–5-variant plus the homozygous ACMG 1 c.1238G>A variant to group 5. However, 5 of 12 patients with homozygous c.1238G>A variant and c.1061C>T variant also had heterozygous c.1391delC variant. Of these, one patient had an abnormal ISTH BS and another patient needed substitution with rFVIIa.

### Genotype/phenotype analyses

For genotype/phenotype analyses, 18 patients (14.7%) with another bleeding disorder besides FVII deficiency (WT *n* = 3, ACMG 1–3 variants, only, *n* = 9, ACMG 4 or 5 variants *n* = 6) were excluded from the assignment to the groups 1 to 5. Of the remaining 111 patients, 22 (19.8%) were *F7* gene wildtype, 29 (26.1%) had ACMG 1 or 2 variants, only, 51 (45.9%) had at least one ACMG 4 or 5 variant, and 9 (8.1%) had to be assigned to group 3 due to ACMG 3 or unclassified variants (Table 2). Only one patient with at least two heterozygote ACMG 4 or 5 variants was known to be compound heterozygote.

**TABLE 2:** Characteristics, ISTH BS, and substitution in genetic subgroups of patients with FVII deficiency according to Figure 1.

	Group 1	Group 2	Group 3	Group 4	Group 5	Group 6
	*F7* gene wildtype	ACMG 1 or ACMG 2	ACMG 3 or not classified ± ACMG 1–2 heterozygous	ACMG 4 or ACMG 5 single heterozygous ± ACMG 1–3 single heterozygous	ACMG 4 or ACMG 5 homozygous or complex heterozygous^a^	FVII deficiency and another bleeding disorder^b^
Number of patients	*n* = 22	*n* = 29	*n* = 9	*n* = 20	*n* = 31	*n* = 18
Number of *F7* gene variants per patient^c^	0	1.1 ± 0.4 1 (1–3)	1.7 ± 1.3 1 (1–5)	1.6 ± 0.5 2 (1–2)	2.7 ± 1.7 2 (1–10)	1.2 ± 0.8 1 (0–3)
Age^d^	36.9 ± 14.1 34 (16–69)	31.9 ± 19.0 25 (1–84)	30.2 ± 15.4 37 (0–48)	34.0 ± 17.5 34.5 (2–82)	36.5 ± 19.3 30 (6–83)	32.4 ± 10.1 32 (6–51)
Female sex	17 (77.3%)	18 (62.1%)	6 (66.7%)	16 (80.0%)	17 (54.8%)	15 (83.3%)
**Reason for consultation**						
- Bleeding symptoms	1 (4.5%)	5 (17.2%)	0	5 (25.0%)	4 (12.9%)	8 (44.4%)
- Laboratory findings	4 (18.1%)	6 (20.7%)	2 (22.2%)	5 (25.0%)	14 (45.2%)	0
- Family screening	3 (13.6%)	6 (20.7%)	1 (11.1%)	2 (10.0%)	0	0
- FVII deficiency known	1 (4.5%)	5 (17.2%)	2 (22.2%)	0	9 (29.0%)	0
- Abortions/wish for child	12 (54.5%)	4 (13.8%)	3 (33.3%)	6 (30.0%)	4 (12.9%)	9 (50.0%)
- Others	1 (4.5%)	3 (10.3%)	1 (11.1%)	2 (10.0%)	0	1 (5.6%)
FVII activity (%)^c^	48.6 ± 14.0 48.5 (8–67)	45.7 ± 14.5 48.0 (8–66)	44.1 ± 11.0 42 (28–63)	43.4 ± 13.6 46 (19–65)	29.5 ± 13.6 30.0 (2–61)	49.7 ± 10.2 49.5 (34–65)
Quick value (%)^c^	74.7 ± 15.0 76 (27–99)	68.2 ± 15.6 72 (21–87)	76.2 ± 12.5 78 (54–98)	70.3 ± 14.1 71.5 (46–90)	54.4 ± 16.4 57 (16–92)	72.3 ± 10.9 72 (53–92)
**ISTH BS^c^**						
- Females	1.5 ± 1.7	1.7 ± 1.5	2.2 ± 1.9	2.3 ± 1.4	3.5 ± 2.7	2.4 ± 1.8
	1 (0–6)	1 (0–5)	1.5 (0–5)	2 (0–5)	3 (0–9)	3 (0–6)
- Males	0.6 ± 0.9	1.3 ± 1.1	1 and 1	0, 0, and 2	1.6 ± 1.4	2 and 8
	0 (0–2)	2 (0–3)			2 (0–5)	
- Children	-	0 and 2	2	0	3 and 5	2
ISTH BS ≥ cut-off	1 (4.5%)	0 (0%)	0 (0%)	0 (0%)	7 (22.6%)	2 (11.1%)
- Females ≥ 6	1/17	0/18	0/6	0/16	4/16	1/15
- Males ≥ 4	0/5	0/9	0/2	0/3	1/13	1/2
- Children ≥ 3	0/0	0/2	0/1	0/1	2/2	0/1
rFVIIa substitution	1 (4.5%)	1 (3.4%)	0 (0%)	1 (5.0%)	5 (16.1%)^e^	3 (16.7%)^f^

BS, bleeding score; rFVIIa, recombinant factor VIIa; SD, standard deviation.

^a^Two patients with heterozygous single ACMG 4 or 5 variant and single ACMG 3 variant included in group 5 due to FVII:C <30%.

^b^The grey color indicates that patients in group 6 were excluded from the assignment to groups 1 to 5 due to another bleeding disorder besides FVII deficiency: von Willebrand syndrome (vWS) (*n* = 10), FV deficiency (*n* = 1), FX deficiency (*n* = 1), FXI deficiency (*n* = 4), PAI-1 deficiency (*n* = 1), FXIII deficiency (*n* = 1).

^c^First row: mean ± SD or numbers in single patients; second row: median (range).

^d^Mean ± SD.

^e^One female with ISTH BS 9; 4 patients with ISTH BS < cut-off.

^f^One female with ISTH BS 6; 2 patients with ISTH BS < cut-off.

Median number of *F7* gene variants per patient was 2 in group 4 (range 1–2) as well as in group 5 (range 1–10) whereas median number was 1 in groups 2 (range 1–3), 3 (range 1 – 5) and 6 (range 0–3) (Table 2). Mean age ranged from 30.2 ± 15.4 years in group 3 (ACMG 3 or not classified) to 36.9 ± 14.1 years in group 1 (*F7* gene wildtype).

The groups differed with respect to the proportion of patients with female sex between 54.8% in group 5 and 83.3% in group 6. Reasons for consultation of the Coagulation Center were also different. Group 6 reached the numerically highest rates in the consultation categories bleeding symptoms (44.4%), and abortions and/or wish for a child (50.0%), the latter together with group 1 (54.5%). Most patients in group 5 were admitted due to abnormal laboratory findings (45.2%) or known FVII deficiency (29.0%) (Table 2).

**FIGURE 2:**
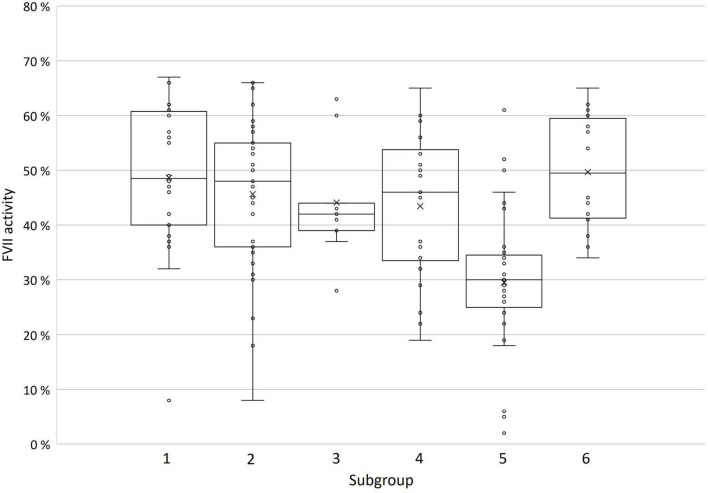
FVII activities stratified by six subgroups according to Figure 1.

Mean FVII:C and Quick values were lowest in group 5 (29.5 and 54.4%, respectively), and ranged from 43.4 to 49.7%, and 68.2 to 76.2% in the other groups, respectively (Figure 2 and Table 2). In group 5, FVII:C was 5–6% in three patients with homozygous ACMG 4 or 5 variants, 26.4 ± 10.7% in 10 patients with ≥2 heterozygous ACMG 4 or 5 variants (*n* = 8) or ACMG 3–5 variants (*n* = 2), and 35.3 ± 11.1% in 18 patients with one heterozygous ACMG 4 or 5 variant combined with homozygous c.1238G>A or ≥2 ACMG 1–3 variants.

In females ≥12 years, mean ISTH BS was numerically higher in group 5 (3.5 ± 2.7; median 3; range 0–9) versus 1.9 ± 1.6 (median 1, range 0–6) in groups 1 to 4 (pooled data of 57 females). In males ≥12 years, 13 patients of group 5 had mean ISTH BS of 1.6 ± 1.4 (median 2, range 0–5) versus 1.0 ± 1.0 (median 1, range 0–3) in 19 males ≥12 years included in groups 1 to 4. Both children included in group 5 had an abnormal ISTH BS versus none of 4 children in groups 1 to 4 (Table 2).

Eleven of 31 pts. (35.5%) in group 5 had an abnormal ISTH-BS (*n* = 7) and/or history of substitution with recombinant factor VIIa (*n* = 5) versus 4 of 80 pts. (5.0%, *n* = 1 abnormal ISTH-BS, *n* = 3 substitution) in groups 1 to 4 (Table 2). In group 5, the percentages of patients with abnormal ISTH-BS and/or history of substitution were similar in patients with homozygous ACMG 4 or 5 variants (1 of 3 patients, 33%), ≥2 heterozygous ACMG 4 or 5 variants (3 of 10 patients, 30%), one heterozygous ACMG 4 or 5 variant plus homozygous c.1238G>A variant (6 of 15 patients, 40%) and one heterozygous ACMG 4 or 5 variant plus ≥2 heterozygous ACMG 1 to 3 variants (1 of 3 patients, 33%). In group 6 (FVII deficiency and another bleeding disorder), 4 of 18 patients (22.2%) had an abnormal ISTH-BS (*n* = 2) and/or history of substitution with recombinant factor VIIa (*n* = 3).

## Discussion

To the best of our knowledge, this exploratory analysis is the first investigation focusing on the usefulness of ACMG classification for interpretation of molecular genetic test results in patients with FVII deficiency. On one site, an abnormal ISTH-BS and/or a history of substitution was observed in few patients (4/80; 5.0%) with *F7* gene wildtype or ACMG 1–3 variants or single heterozygous ACMG 4–5 variant with/without single heterozygous ACMG 1–3 variant. On the other site, 11 of 31 patients (35.5%) with four different scenarios of ACMG 4–5 ± ACMG 1–3 variant findings reached the combined endpoint of this exploratory analysis. Pooling patients with different constellations of zygosity and ACMG classifications in two genotypic categories was based on our preceding analysis of 44 patients with co-existence of heterozygous or homozygous c.1238G>A variant and heterozygous ACMG 5 c.1061C>T variant which was not biased by possible differences between variants of the same ACMG class such as truncating versus not-truncating variations (6) or large deletions versus others (27). This preceding analysis supports the hypothesis, that heterozygous ACMG 4–5 variants may become clinically relevant if combined with a modifying homozygous ACMG 1–3 variant or ≥2 heterozygous ACMG 1–3 variants. In the total cohort, 7 of 18 patients (38.9%) with this kind of molecular genetic finding reached the combined end point which is similar to 4 of 13 patients (30.8%) with homozygous or compound/double heterozygous ACMG 4 or 5 variants. Homozygous and compound heterozygous pathogenic *F7* variants are known to be associated with an increased bleeding risk (2, 6, 28). Of note, differentiation between double heterozygosity affecting one allele, only, and compound heterozygosity was not available in 9 of 10 patients with two heterozygous ACMG 3–5 variants in our cohort.

Since 2015, the ACMG classification has been introduced as the internationally accepted standard for the interpretation of gene variants in heritable diseases (15, 16). For example, novel *F8* and *F9* gene variants in patients with hemophilia A or B included in the PedNet Registry were classified according to ACMG/AMP guidelines (29). In children with suspected inherited bleeding disorder genetic screening based on the ACMG guidelines may serve as a tool for early diagnosis, especially in patients with inherited thrombocytopenia (30). In the EAHAD blood coagulation factor VII variant database the assignment of *F7* gene variants to ACMG classes is already foreseen (11). Based on the ACMG classification a simplification may be possible by assigning patients with FVII deficiency and no other bleeding disorder to the categories “low risk” and “high risk” for abnormal bleeding tendency.

Our investigation may serve as a kick-off to promote the use of ACMG classification in FVII deficiency. One major question to be answered in patients with FVII deficiency refers to prophylaxis with procoagulants for surgery (1) or for delivery (31). In the past, the prediction of bleeding risk to decide on substitution therapy has been mainly based on FVII:C levels, the personal clinical history and the first bleeding symptom (1, 32). In a larger cohort and preferably prospective study, the hypothesis may be tested that the two categories identified in our investigation (“low risk” versus “high risk” for abnormal bleeding tendency) are appropriate to support ACMG classification-based interpretation of molecular genetic test results in FVII deficient patients as a contribution to successive advice and decision making in daily routine.

Even in the “high risk” group the median FVII:C was rather high (30.0%, range 2–61%) if compared to an Italian investigation which showed FVII:C > 25% in 7.1% of homozygous or compound heterozygous patients, only (6). Whereas the Italian study included patients with FVII:C < 50% diagnosed because of bleeding tendency or prolonged prothrombin time, only, maximum FVII:C was higher in our retrospective study and other reasons for consultation such as family screening or abortions/wish for a child were allowed.

As already shown by the Greifswald FVII deficiency study, the most frequent pathogenic variant in the German population is the ACMG 5 missense mutation p.(Ala294Val) (c.1061C>T) (33), also frequently combined with the ACMG 5 variant c.1391delC p.(Pro464Hisfs*32) (34) which was the same in Polish and Italian cohorts (35, 36). In our cohort the c.1061C>T variant was present in 19 of 129 patients (14.7%). Six of these patients had a combination with the c.1391delC variant and 18 patients with the c.1238G>A modifying variant. As published by Fromovich-Amit et al. (23) FVII secretion was not further reduced by the variant c.1238G>A in the presence of the c.1061C>T variant. In our cohort there was a small difference with respect to FVII activity in patients heterozygous for c.1061C>T variant together with homozygous c.1238G>A variant (FVII:C 32.6 ± 5.8%, *n* = 12) versus combination with heterozygote c.1238G>A variant (FVII:C 38.3 ± 11.1%, *n* = 6) (Table 1).

We observed a difference in zygosity of ACMG 1 or 2 variants versus ACMG 4 or 5 variants (homozygous: 52.9% versus 4.6%). This may reflect the typically higher minor allele frequency (MAF) of ACMG 1 or 2 variants versus ACMG 4 or 5 variants in conjunction with the higher probability of decreased FVII:C in homozygous versus heterozygous variants.

Key limitations of this investigation are the retrospective design based on patient datasets and some ACMG findings derived from partially outdated rules for classification of pathogenicity and older literature searches. It should be noted that the ACMG classification is a learning system with recent or upcoming new recommendations (37). A disease specific expert panel has been founded for coagulation factor deficiencies (38). However, until now, published experiences to adapt criteria for ACMG classification are available for other diseases such as recessively inherited hemoglobinopathies, only (39). Further limitations of our analyses are the exclusion of patients without molecular genetic testing, the baseline differences between groups, no FVII antigen testing to evaluate possible FVII:C discrepancies (40), and restriction to descriptive statistics without *p*-values as no formal hypothesis was defined in advance.

In conclusion, ACMG classification is a promising tool to improve interpretation of molecular genetic test results in patients with FVII deficiency. The results of this exploratory analysis suggest phenotype differences between patients with a homozygous ACMG 4–5 variant or specific combinations of heterozygous ACMG 4–5 ± ACMG 1–3 variants, on one side, and patients with *F7* gene wildtype, ACMG 1–3 variants, only, or single heterozygous ACMG 4–5 ± single heterozygous ACMG 1–3 variants, on the other side. This finding may serve as a basis to develop a genotype/phenotype prediction model in future studies.

## Data availability statement

The datasets presented in this study can be found in online repositories. The names of the repository/repositories and accession number(s) can be found in the article and Supplementary Tables 1–3.

## Author contributions

RA had the idea and concept for the study and did the preparing work as well as manuscript outline. CH and C-ED helped to detect the patients, gave their input to the idea, and did proofreading. BR worked in the lab and did essential work to find out the ACMG classification. DJ performed the statistical analysis, literature and *F7* variant database reviews, and was involved in the preparation of the manuscript. All authors contributed to the article and approved the submitted version.

## Footnotes

1
https://gnomad.broadinstitute.org

2
https://www.ncbi.nlm.nih.gov/clinvar/

3
https://varsome.com

4
https://franklin.genoox.com

5
https://f7-db.eahad.org
