# Supplementary material for: Utility of ACMG classification to support interpretation of molecular genetic test results in patients with factor VII deficiency
Source: Front Med (Lausanne). 2023 Jul 14;10:1220813. doi: 10.3389/fmed.2023.1220813 (PMC10382174; doi:10.3389/fmed.2023.1220813)
Supplement: Supplementary file 1 [file Table_1.pdf]

Table 1: ACMG 1 or ACMG 2 variants found in a cohort of patients with FVII deficiency and molecular genetic testing

Region	cDNA change c. …	Number pts. in cohort			EAHAD database [https://f7-db.eahad.org (accessed Dec 20, 2022)]									
		Total	Homo-zygous	Single variant	Variant number	Pts. (n)	MAF	Grant-ham Score	PolyPhen-2 Prediction		SIFT Prediction		PROVEAN Prediction	
									Score	Damaging?	Score	Category	Score	Category
**ACMG 1**														
5'UTR	-336_-335[10] or -232ins10	1	0	0	-	0								
Flanking (5')	-122T>C	4	2	0	995	6	0.1436							
Intron 1	64+9G>A	1	1	0	993	7	0.1404							
Exon 6	525C>T	4	2[a]	0	999	63	0.1346							
Exon 9	1238G>A	81	47	35	998	132	0.1265	43	0.67	Possibly	0.132	Tolerated	-1.16	Neutral
Exon 9, 3'UTR	*770G>A	3	1	0	-	0								
Exon 9, 3'UTR	*639G>A	1	0	0	-	0								
Exon 9, 3'UTR	*673A>G	1	0	0	-	0								
Exon 9, 3'UTR	*1146A>G	1	0	0	-	0								
Exon 9, 3'UTR	*1275A>G	1	0	0	-	0								
**ACMG 2**														
Exon 3	150A>C	1	0	0	-	0								
Exon 9, 3'UTR	*481_*482delAG	2	1	0	-	0								
Intron 8	805+7A>G	1	0	0	73	11	0.0038							
**Sum**		**102**	**54 (53%)[a]**	**35 (35%)**		**219**								

[a]n=1 missing information
