# Supplementary material for: Utility of ACMG classification to support interpretation of molecular genetic test results in patients with factor VII deficiency
Source: Front Med (Lausanne). 2023 Jul 14;10:1220813. doi: 10.3389/fmed.2023.1220813 (PMC10382174; doi:10.3389/fmed.2023.1220813)
Supplement: Supplementary file 2 [file Table_2.pdf]

## Supplementary Table 2: ACMG 3 or not classified variants found in a cohort of patients with FVII deficiency and molecular genetic testing

| Region | cDNA change c. … | Number pts. in cohort | | | EAHAD database [https://f7-db.eahad.org (accessed Dec 20, 2022)] | | | | | | | | | |
|---|---|---|---|---|---|---|---|---|---|---|---|---|---|---|
| | | Total | Homo-zygous | Single variant | Variant number | Pts. (n) | MAF | Grant-ham Score | PolyPhen-2 Prediction | | SIFT Prediction | | PROVEAN Prediction | |
| | | | | | | | | | Score | Damaging? | Score | Category | Score | Category |
| **ACMG 3** | | | | | | | | | | | | | | |
| 5'UTR | 1-95C>T | 2 | 0 | 1 | - | 0 | | | | | | | | |
| 5'UTR | -44T>C | 1 | 0 | 0 | - | 0 | | | | | | | | |
| Exon 1 | 56T>C | 1 | 0 | 0 | 12 | 4 | 4.70E-6 | 98 | 0.98 | Probably | 0.000 | Damaging | -1.76 | Neutral |
| Intron 1 | 65-3C>T | 1 | 1 | 0 | 201 | 3 | 7.61E-4 | | | | | | | |
| Exon 5 | 416G>A | 1 | 1 | 0 | 41 | 7 | 6.05E-4 | 43 | 1.00 | Probably | 0.065 | Tolerated | -2.14 | Neutral |
| Intron 7 | 682-3C>G | 1 | 0 | 1 | - | 0 | | | | | | | | |
| Exon 8 | 725T>C | 1 | 0 | 1 | - | 0 | | | | | | | | |
| Exon 8 | 806-10T>C | 1 | 0 | 0 | - | 0 | | | | | | | | |
| Exon 9 | 1211G>A | 1 | 0 | 1 | - | 0 | | | | | | | | |
| Exon 9 | 1264G>T | 1 | 0 | 1 | 159 | 1 | | 50 | 1.00 | Probably | 0.001 | Damaging | -4.63 | Deleterious |
| **Sum** | | **11** | **2 (18%)** | **5 (45%)** | | **15** | | | | | | | | |
| **Not classified** | | | | | | | | | | | | | | |
| 5'UTR | -51-4C>G | 1 | 0 | 1 | - | 0 | | | | | | | | |
| Exon 9, 3'UTR | *153_*154insAA | 3 | 2 | 0 | - | 0 | | | | | | | | |
| **Sum** | | **4** | **2 (50%)** | **1 (25%)** | | **0** | | | | | | | | |
