# Supplementary material for: Utility of ACMG classification to support interpretation of molecular genetic test results in patients with factor VII deficiency
Source: Front Med (Lausanne). 2023 Jul 14;10:1220813. doi: 10.3389/fmed.2023.1220813 (PMC10382174; doi:10.3389/fmed.2023.1220813)
Supplement: Supplementary file 3 [file Table_3.pdf]

Supplementary Table 3: ACMG 4 or ACMG 5 variants found in a cohort of patients with FVII deficiency and molecular genetic testing

Region	cDNA change c. …	Number pts. in cohort			EAHAD database [https://f7-db.eahad.org (accessed Dec 20, 2022)]									
		Total	Homo-zygous	Single variant	Variant number	Pts. (n)	MAF	Grant-ham Score	PolyPhen-2 Prediction		SIFT Prediction		PROVEAN Prediction	
									Score	Damaging?	Score	Category	Score	Category
**ACMG 4**														
Exon 3	152C>A	1	0	0	167	1		126	0.99	Probably	0.000	Damaging	-5.32	Deleterious
Exon 3	262C>G	1	0	1	25	3	1.79E-5	125	0.99	Probably	0.001	Damaging	-5.93	Deleterious
Exon 6	469G>A	1	0	0	46	5	2.14E-5	56	1.00	Probably	0.068	Tolerated	-3.25	Deleterious
Exon 8	751G>A	1	0	0	67	6		58	1.00	Probably	0.000	Damaging	-3.77	Deleterious
Exon 9	934G>A	4	0	0	83	25	6.41E-5	21	1.00	Probably	0.003	Damaging	-2.12	Neutral
Exon 9	1009C>T	1	0	0	88	13	1.03E-4	180	1.00	Probably	0.011	Damaging	-5.83	Deleterious
Exon 9	1151C>T	1	1	0	147	9	7.84E-5	81	1.00	Probably	0.005	Damaging	-5.28	Deleterious
Exon 9	1160T>C	1	0	1	-	0								
Exon 9	1311C>G	1	0	1	-	0								
Exon 9	1384C>T	1	0	0	165	6	3.19E-5							
Exon 9	1388delC	1	0	0	-	0								
**ACMG 5**														
Exon 1	64G>A	1	1	1	13	4	1.45E-5	56	0.97	Probably	0.070	Tolerated	0.20	Neutral
Exon 3	211G>A	1	0	0	-	0								
Intron 3	291+1G>A	2	0	1	27	6								
Intron 5	430+1G>A	1	0	1	42	15	1.99E-5							
Exon 6	479A>G	3	0	0	49	60	9.61E-5	43	1.00	Probably	0.010	Damaging	-2.78	Deleterious
Exon 7	583T>C	2	0	1	55	16	4.0E-6	180	1.00	Probably	0.013	Damaging	-11.16	Deleterious
Exon 7	635G>A	1	0	0	58	13	8.0E-6	43	1.00	Probably	0.001	Damaging	-3.01	Deleterious
Exon 7	647delG	2	0	2	59	1	4.0E-6							
Exon 9	817-831del	1	0	1	262	1	8.4E-6							
Exon 9	911C>T	2	1	1	80	58	1.50E-4	64	0.99	Probably	0.018	Damaging	-3.61	Deleterious
Exon 9	920G>A	1	0	0	82	2	2.5E-5	29	0.49	Possibly	0.088	Tolerated	-2.74	Deleterious
Exon 9	1027G>A	1	0	0	91	12	7.1E-6	56	1.00	Probably	0.000	Damaging	-5.85	Deleterious
Exon 9	1061C>T	19	0	0	94	117	5.61E-4	64	0.94	Possibly	0.003	Damaging	-3.12	Deleterious
Exon 9	1091G>A	1	0	0	101	72	4.85E-4	43	1.00	Probably	0.054	Tolerated	-2.09	Neutral
Exon 9	1109G>T	6	0	1	102	45	6.43E-5	205	1.00	Probably	0.000	Damaging	-8.75	Deleterious
Exon 9	1247G>A	1	0	0	117	5								
Exon 9	1391delC	6	0	0	125	63	6.74E-5							
**Sum**		**65**	**3 (5%)**	**12 (18%)**		**558**								
