# Supplementary material for: Utility of ACMG classification to support interpretation of molecular genetic test results in patients with factor VII deficiency
Source: Front Med (Lausanne). 2023 Jul 14;10:1220813. doi: 10.3389/fmed.2023.1220813 (PMC10382174; doi:10.3389/fmed.2023.1220813)
Supplement: Supplementary file 4 [file Table_4.pdf]

No.	Sub-group	Age class	Number *F7* variants	*F7* variants	c.1238G>A variant homoz/ heteroz	Other variants homoz/ heteroz	ISTH-bleeding score	FVII:C	Quick value (%)	rFVIIa substi-tution 0=no 1=yes
1	6	≥12	1	Exon 9 c.1238G>A homoz ACMG 1	homoz		6	44%	69	1
2	1	≥12	0	Wildtype			1	48%	82	0
3	5	≥12	2	5'UTR c.1-95C>T ACMG 3, Exon 3 c.211G>A ACMG 5, compound heteroz		comp. heteroz	1	22%	29	0
4	3	<12	1	5'UTR c.1-95C>T heteroz ACMG 3		heteroz	2	41%	98	0
5	6	≥12	1	Exon 9 c.1238G>A homoz ACMG 1	homoz		3	36%	64	0
6	5	≥12	2	Exon 9 c1238G>A homoz ACMG 1, Exon 9 c.911C>T homoz ACMG 5	homoz	homoz	2	6%	22	0
7	5	<12	2	Exon 9 c.1238G>A homoz ACMG 1, Exon 9 c.1061C>T heteroz ACMG 5	homoz	heteroz	5	36%	59	0
8	1	≥12	0	Wildtype			0	42%	69	0
9	4	≥12	2	Exon 9 c.1238G>A heteroz ACMG 1, Exon 9 c.1061C>T heteroz ACMG 5.	heteroz	heteroz	0	53%	81	0
10	3	≥12	1	Exon 9 c.1211G>A heteroz ACMG 3		heteroz	5	37%	69	0
11	3	≥12	1	Exon 8 c.725T>C heteroz ACMG 3		heteroz	1	28%	54	0
12	4	≥12	2	Exon 9 c.1238G>A heteroz ACMG 1, Exon 9 c.1247G>A heteroz ACMG 5	heteroz	heteroz	1	19%	46	0
13	1	≥12	0	Wildtype			0	57%	84	0
14	3	≥12	2	Exon 9 c.1238G>A heteroz ACMG 1, Exon 3 c.150A>C heteroz ACMG 3,	heteroz	heteroz	2	43%	83	0
15	5	≥12	2	Exon 9 c.1238G>A homoz ACMG 1, Exon 9 c.1061C>T heteroz ACMG 5	homoz	heteroz	8	31%	58	0
16	6	≥12	0	Wildtype			3	61%	88	0
17	1	≥12	0	Wildtype			0	60%	62	0
18	2	≥12	1	Exon 9 c.1238G>A homoz ACMG 1	homoz		5	53%	72	0
19	5	≥12	2	Exon 9 c.1238G>A homoz ACMG 1, Exon 9 c.1061C>T heteroz ACMG 5	homoz	heteroz	0	30%	56	0
20	2	≥12	1	Exon 9 c.1238G>A homoz ACMG 1	homoz		0	31%	51	0
21	2	≥12	1	Exon 9 c.1238G>A homoz ACMG 1	homoz		0	48%	80	0

22	5	≥12	3	Exon 9 c.1238G>A heteroz ACMG 1, Flanking (5') c.1-122T heteroz ACMG1, Exon 7 c.635G>A heteroz ACMG 5	heteroz	heteroz + heteroz	6	34%	47	0
23	1	≥12	0	Wildtype			2	62%	96	0
24	1	≥12	0	Wildtype			1	61%	99	0
25	1	≥12	0	Wildtype			0	66%	80	0
26	6	<12	1	Exon 9 c.1238G>A heteroz ACMG 1	heteroz		2	54%	86	0
27	4	≥12	2	Exon 9 c.1238G>A heteroz ACMG 1, Exon 9 c.1061C>T heteroz ACMG 5	heteroz	heteroz	2	37%	61	0
28	2	≥12	1	Exon 9 c.1238G>A homoz ACMG 1	homoz		2	53%	72	0
29	4	≥12	1	Intron 5 c.430+1G>A heteroz ACMG 5		heteroz	1	46%	80	0
30	2	≥12	1	Exon 9 c.1238G>A heteroz ACMG 1	heteroz		1	18%	48	0
31	2	<12	2	Exon 9 c.1238G>A homoz ACMG 1, Flanking(5') c.1-122T>C homoz ACMG 1	homoz	homoz	2	23%	40	0
32	1	≥12	0	Wildtype			0	8%	27	1
33	5	≥12	3	Exon 9 c.1238G>A heteroz ACMG 1, Exon 9 c.1061C>T heteroz ACMG 5, Exon 9 c.1391delC heteroz ACMG 5	heteroz	heteroz + heteroz	3	19%	53	0
34	4	≥12	1	Exon 7 c.647delG heteroz ACMG 5		heteroz	3	59%	90	0
35	6	≥12	2	Exon 9 c.1238G>A heteroz ACMG 1, Intron1 c.65-3C>T homoz ACMG 3	heteroz	homoz	1	57%	83	0
36	1	≥12	0	Wildtype			1	49%	69	0
37	1	≥12	0	Wildtype			3	67%	84	0
38	2	≥12	1	Exon 9 c.1238G>A homoz ACMG 1	homoz		0	59%	78	0
39	1	≥12	0	Wildtype			6	36%	69	0
40	5	≥12	10	Exon 9 c.1238G>A heteroz ACMG 1, Exon 9 c.1061C>T heteroz ACMG 5, Exon 6 c.525C>T heteroz ACMG 1, Exon 9 3'UTR c.*153_*154insAA homoz (not classified), Exon 9 3'UTR c.*481_*482delAG ACMG 2, Exon 9 3'UTR c.*639G>A heteroz ACMG 1, Exon 9 3'UTR c.*673A>G heteroz ACMG 1, Exon 9 3'UTR c.*770G>A heteroz ACMG 1, Exon 9 3'UTR c.*1146A>G heteroz ACMG 1, Exon 9 3'UTR c.*1275A>G heteroz ACMG 1	heteroz	1x homoz, 8x heteroz	2	31%	57	0

41	5	≥12	2	5'UTR c.-44T>C heteroz ACMG 3, Exon 9 c.1009C>T heteroz ACMG 4		heteroz + heteroz	4	29%	57	0
42	5	≥12	3	Exon 9 c.1238G>A homoz ACMG 1, Exon 9 c.1061C>T heteroz ACMG 5, Exon 9 c.1391delC heteroz ACMG 5	homoz	heteroz + heteroz	3	29%	60	0
43	1	≥12	0	Wildtype			0	40%	62	0
44	5	≥12	2	Exon 9 c.1238G>A homoz ACMG 1, Intron 3 c.291+1G>A heteroz ACMG 5	homoz	heteroz	1	28%	62	1
45	5	<12	2	Exon 9 c.1238G>A homoz ACMG 1, Exon 9 c.1061C>T heteroz ACMG 5	homoz	heteroz	3	28%	57	0
46	2	≥12	1	Exon 9 c.1238G>A heteroz ACMG 1	heteroz		3	53%	81	0
47	5	≥12	4	Exon 9 c.1238G>A heteroz ACMG 1, Exon 9 c.1091G>A heteroz ACMG 5, 5'UTR c.-336_-335 (10) (alternative nomenclature: c.-232ins10) heteroz ACMG 1, Flanking(5') c.1-122T>C heteroz ACMG 1	heteroz	heteroz + heteroz + heteroz	2	50%	59	0
48	1	≥12	0	Wildtype			2	38%	76	0
49	1	≥12	0	Wildtype			1	40%	75	0
50	4	≥12	1	Exon 9 c.817-831del heteroz ACMG 5		heteroz	4	50%	66	0
51	4	<12	2	Exon 9 c.1238G>A heteroz ACMG 1, Exon 9 c.1109G>T heteroz ACMG 5	heteroz	heteroz	0	22%	59	0
52	5	≥12	3	Exon 9 c.1238G>A homoz ACMG 1, Exon 9 c.1061C>T heteroz ACMG 5, Exon 9 c.1391delC heteroz ACMG 5	homoz	heteroz + heteroz	0	27%	57	0
53	5	≥12	2	Exon 9 c.1238G>A homoz ACMG 1, Exon 9 c.934G>A heteroz ACMG 4	homoz	heteroz	5	46%	71	0
54	6	≥12	0	Wildtype			1	41%	92	0
55	5	≥12	2	Exon 9 c.1238G>A homoz ACMG 1, Exon 9 c.934G>A heteroz ACMG 4	homoz	heteroz	2	33%	71	1
56	4	≥12	1	Intron 3 c.291+1G>A heteroz ACMG 5		heteroz	0	34%	63	0
57	4	≥12	2	Exon 9 c.1238G>A heteroz ACMG 1, Exon 9 c.1027G>A heteroz ACMG 5	heteroz	heteroz	1	56%	75	0
58	1	≥12	0	Wildtype			2	37%	66	0
59	6	≥12	2	Exon 9 c.1238G>A heteroz ACMG 1, Exon 9 c.920G>A heteroz ACMG 5	heteroz	heteroz	2	41%	53	0
60	2	≥12	1	Exon 9 c.1238G>A homoz ACMG 1	homoz		0	37%	73	0
61	2	≥12	1	Exon 9 c.1238G>A heteroz ACMG 1	heteroz		0	30%	52	0

62	6	≥12	1	Exon 9 c.1238G>A heteroz ACMG 1	heteroz		0	54%	66	0
63	3	≥12	2	Exon 9 c.1238G>A heteroz ACMG 1, Exon 1, c.56T>C heteroz ACMG 3	heteroz	heteroz	1	39%	70	0
64	4	≥12	1	Exon 3 c.262C>G heteroz ACMG 4		heteroz	4	51%	78	0
65	2	≥12	1	Exon 9 c.1238G>A homoz ACMG 1	homoz		2	36%	58	0
66	1	≥12	0	Wildtype			0	46%	68	0
67	2	≥12	1	Exon 9 c.1238G>A homoz ACMG 1	homoz		0	47%	69	0
68	2	≥12	1	Exon 9 c.1238G>A homoz ACMG 1	homoz		1	42%	61	0
69	5	≥12	2	Exon 9 c.1238G>A homoz ACMG 1, Exon 9 c. 934G>A heteroz ACMG 4	homoz	heteroz	2	18%	42	0
70	1	≥12	0	Wildtype			3	62%	83	0
71	1	≥12	0	Wildtype			2	55%	86	0
72	5	≥12	2	Exon 9 c.1238G>A homoz ACMG 1, Exon 9 c.1061C>T heteroz ACMG 5	homoz	heteroz	1	44%	66	1
73	5	≥12	2	Exon 5 c.416G>A homoz ACMG 3, Exon c.1151C>T homoz ACMG 4		homoz + homoz	0	5%	31	0
74	2	≥12	1	Exon 9 c.1238G>A homoz ACMG 1	homoz		1	33%	58	0
75	3	≥12	5	Exon 9 c.1238G>A heteroz ACMG 1, Exon 6 c.525C>T heteroz ACMG 1, Intron 8 c.805+7A>G heteroz ACMG 2, Exon 9 UTR c.*153_*154insAA heteroz ACMG 3, Exon 9 UTR c.*770G>A heteroz ACMG 1	heteroz	heteroz + heteroz + heteroz + heteroz	1	44%	80	0
76	3	≥12	1	Exon 9 c.1264G>T heteroz ACMG 3		heteroz	4	63%	69	0
77	5	≥12	3	Exon 9 c.1238G>A homoz ACMG 1, Exon 9 c.1061C>T heteroz ACMG 5, Exon 9 c.1391delC heteroz ACMG 5	homoz	heteroz + heteroz	6	43%	64	0
78	2	<12	1	Exon 9 c.1238G>A homoz ACMG 1	homoz		0	45%	76	0
79	2	≥12	1	Exon 9 c.1238G>A homoz ACMG 1	homoz		3	8%	21	0
80	1	≥12	0	Wildtype			1	32%	61	0
81	1	≥12	0	Wildtype			0	61%	86	0
82	5	≥12	2	Exon 9 c.1238G>A homoz ACMG 1, Exon 9 c.1061C>T heteroz ACMG 5	homoz	heteroz	1	28%	56	0
83	2	≥12	1	Exon 9 c.1238G>A heteroz ACMG 1	heteroz		2	57%	87	1
84	5	≥12	2	Exon 9 c.1061C>T heteroz ACMG 5, Exon 9 c.1391delC heteroz ACMG 5		heteroz + heteroz	5	33%	63	0

85	5	≥12	7	[illegible] Exon 6 c.525C>T ACMG 1, Exon 9 c.1061C>T heteroz ACMG 5, Exon 9 c.1388delC heteroz ACMG 4, Exon 9 3'UTR c.*153_*154insAA homoz (not classified), Exon 9 3'UTR c.*481_*482delAG homoz ACMG 2, Exon 9 3'UTR c.*770G>A homoz ACMG 1	homoz	heteroz + heteroz + homoz + homoz + homoz + missing	2	30%	60	1
86	5	≥12	3	Exon 9 c.1238G>A homoz ACMG 1, Flanking(5') c.1-122T>C homoz ACMG 1, Exon 6 c.479A>G heteroz ACMG 5	homoz	homoz + hetero	3	26%	57	0
87	2	≥12	3	Exon 9 c.1238G>A homoz ACMG 1, Intron 1 c.64+9G>A homoz ACMG 1, Exon 6 c.525C>T homoz ACMG 1	homoz	homoz + homoz	1	50%	57	0
88	5	≥12	2	Exon 6 c.479A>G heteroz ACMG 5, Exon 7 c.583T>C heteroz ACMG 5		heteroz + heteroz	1	2%	16	0
89	6	≥12	1	Exon 9 c.1238G>A homoz ACMG 1	homoz		3	42%	68	0
90	5	≥12	2	Exon 9 c.1238G>A homoz ACMG 1, Exon 9 c.934G>A heteroz ACMG 5	homoz	heteroz	1	24%	50	0
91	3	≥12	1	c.-51-4C>G im 5'UTR heteroz, not classified		heteroz	0	42%	78	0
92	2	≥12	1	Exon 9 c.1238G>A homoz ACMG 1	homoz		3	54%	80	0
93	5	≥12	1	Exon 1 c.64G>A homoz ACMG 5		homoz	9	5%	20	1
94	1	≥12	0	Wildtype			0	47%	76	0
95	5	≥12	3	Exon 9 c.1238G>A homoz ACMG 1, Exon 9 c.1061C>T heteroz ACMG 5, Exon 9 c.1391delC heteroz ACMG 5	homoz	heteroz + heteroz	1	30%	61	0
96	6	≥12	1	Exon 9 c.1238G>A heteroz ACMG 1	heteroz		1	65%	76	0
97	6	≥12	3	Exon 9 c.1238G>A heteroz ACMG1, Exon 8 c.806-10T>C heteroz ACMG 3, Exon 9 c.1061C>T heteroz ACMG 5	heteroz	heteroz + heteroz	2	45%	60	0
98	4	≥12	1	Exon 9 c.1109G>T heteroz ACMG 5		heteroz	3	60%	78	0
99	2	≥12	1	Exon 9 c.1238G>A homoz ACMG 1	homoz		4	44%	63	0
100	4	≥12	2	Exon 9 c.1238G>A heteroz ACMG 1, Exon 6 c.479A>G heteroz ACMG 5	heteroz	heteroz	0	29%	48	0
101	3	≥12	1	Intron 7 c.682-3C>G heteroz ACMG 3		heteroz	1	60%	85	0
102	6	≥12	1	Exon 7 c.583T>C heteroz ACMG 5		heteroz	3	42%	68	0
103	6	≥12	1	Exon 9 c.911C>T heteroz ACMG 5		heteroz	0	58%	82	0

104	6	≥12	2	Exon 9 c.1238G>A homoz ACMG 1, Exon 9 c.1109G>T heteroz ACMG 5	homoz	heteroz	3	60%	75	0
105	2	≥12	1	Exon 9 c.1238G>A heteroz ACMG 1	heteroz		1	66%	78	0
106	4	≥12	2	Exon 9 c.1238G>A heteroz ACMG 1, Exon 9 c.1109G>T heteroz ACMG 5	heteroz	heteroz	1	32%	54	0
107	5	≥12	2	Exon 9 c.1238G>A homoz ACMG 1, Exon 9 c.1109G>T heteroz ACMG 5	homoz	heteroz	3	61%	92	0
108	4	≥12	1	Exon 9 c.1160T>C heteroz ACMG 4		heteroz	3	56%	86	0
109	2	≥12	1	Exon 9 c.1238G>A heteroz ACMG 1	heteroz		3	58%	83	0
110	4	≥12	1	Exon 7 c.647delG heteroz ACMG 5		heteroz	2	65%	90	0
111	5	≥12	2	Exon 9 c.1238G>A homoz ACMG 1, Exon 9 c.1109G>T heteroz ACMG 5	homoz	heteroz	3	52%	75	0
112	6	≥12	2	Exon 9 c.1238G>A heteroz ACMG 1, Exon 9 c.1384C>T heteroz ACMG 4	heteroz	heteroz	4	38%	64	1
113	4	≥12	2	Exon 9 c.1238G>A heteroz ACMG 1, Exon 3 c.152C>A heteroz ACMG 4	heteroz	heteroz	2	24%	53	0
114	6	≥12	0	Wildtype			1	34%	58	0
115	1	≥12	0	Wildtype			4	56%	83	0
116	4	≥12	2	Exon9, c.1238G>A heteroz ACMG 1, Exon6 c.469G>A heteroz ACMG 4	heteroz	heteroz	2	36 %	62	0
117	2	≥12	1	Exon 9 c.1238G>A homoz ACMG 1	homoz		3	55 %	75	0
118	2	≥12	1	Exon 9 c.1238G>A homoz ACMG 1	homoz		0	51 %	72	0
119	4	≥12	2	Exon 9 c.1238G>A heteroz ACMG 1, Exon 8 c.751G>A heteroz ACMG 4	heteroz	hetero	5	46 %	68	1
120	2	≥12	1	Exon 9 c.1238G>A homoz ACMG 1	homoz		1	35 %	67	0
121	4	≥12	1	Exon9 c.1311C>G heteroz ACMG 4		heteroz	2	49 %	89	0
122	6	≥12	1	Exon 9 c.1238G>A heteroz ACMG 1	heteroz		8	62 %	75	0
123	2	≥12	1	Exon 9 c.1238G>A heteroz ACMG 1	heteroz		1	66%	87	0
124	6	≥12	1	Exon 9 c.1238G>A homoz ACMG 1	homoz		5	60%	75	1
125	2	≥12	1	Exon 9 c.1238G>A homoz ACMG 1	homoz		2	45 %	67	0
126	2	≥12	1	Exon 9 c.1238G>A heteroz ACMG 1	heteroz		2	62%	87	0
127	4	≥12	2	Exon9 c.1238G>A heteroz ACMG 1, Exon9, c.1061C>T heteroz ACMG 5	heteroz	heteroz	3	45%	78	0
128	5	≥12	2	Exon 9 c.1238G>A homoz ACMG 1, Exon 9 c.1061C>T heteroz ACMG 5	homoz	heteroz	0	35%	58	0
129	2	≥12	1	Exon 9 c.1238G>A homoz ACMG 1	homoz		1	65%	85	0
